# Retrospective analysis of cervical transforaminal versus interlaminar epidural steroid injections

**DOI:** 10.1016/j.inpm.2022.100102

**Published:** 2022-05-25

**Authors:** Josh Levin, John Chan, Nolan Gall, Jayme Koltsov, Lisa Huynh

**Affiliations:** a Department of Orthopaedic Surgery, Stanford University, United States; b Department of Neurosurgery, Stanford University, United States

**Keywords:** Cervical, Epidural, Transforaminal, Interlaminar, Injection

## Abstract

**Background:**

Several studies have compared outcomes from lumbar epidural steroid injections (ESIs) based upon technique (transforaminal (TF) vs interlaminar (IL) vs caudal). However, little on this topic has been reported in the cervical spine, and results have been conflicting.

**Purpose:**

To compare success rates of fluoroscopically-guided cervical TFESIs vs ILESIs.

**Study design/setting:**

Retrospective, observational, in vivo study of consecutive patients at outpatient Physical Medicine & Rehabilitation clinics at a single academic spine center.

**Patient sample:**

Consecutive patients who received a cervical TF or IL ESI between January 2010 and October 2018.

**Outcome measures:**

NRS pain scores within 60 days of the ESI.

**Methods:**

Current procedural terminology (CPT) codes were used to search all consecutive patients at a single outpatient academic spine center who received a cervical TF or IL ESI between January 2010 October 2018. All patients with pre and post injection NRS pain scores within 60 days of the injection were included in the analysis.

**Results:**

A total of 178 ​TF and 185 ILESIs were analyzed. Success was defined as ≥ 50% improvement in NRS pain score. 52% [95% CI: 47 – 57%] of all patients receiving a cervical ESI achieved a successful outcome. There was a strong trend towards better results in the ILESI group with 59% [95% CI: 52 – 66%] of patients achieving at least 50% pain relief compared to 46% [95% CI: 39 – 53%] in the TF group. A higher proportion of patients in the IL group obtained at least 80% pain relief (37% [95% CI: 30 – 44%]) compared to those in the TF group (17% [95% CI: 11 – 23%]). Post-procedure median NRS pain scores, and improvement in median NRS pain scores were better in the ILESI group compared to the TFESI group (p<0.001).

**Conclusion:**

This retrospective study demonstrated better results in the cervical ILESI group compared to the cervical TFESI group.

## 1 Introduction

Cervical epidural steroid injections (ESIs) are targeted interventions that are commonly performed in the treatment of cervical radicular pain. These injections were first reported in the 1930s [1]. The procedure was performed using this technique exclusively for several decades until the transforaminal technique gained popularity [2]. Despite excitement for this new technique, concerns arose due to reports of serious complications and deaths [3]. However, reports of serious complications and deaths from cervical ILESIs were also published [[4], [5], [6], [7]] [[4], [5], [6], [7]] [[4], [5], [6], [7]], and some questioned the growing conventional wisdom that ILESIs were safer than TFESIs [8]. Since a consensus has not been reached regarding the relative safety of the two techniques, the question of relative effectiveness has gained importance.

In contrast to the cervical spine, there are numerous studies comparing the effectiveness of different routes of ESIs in the lumbar spine. For patients with a lumbar disc herniation, TFESI is superior to ILESI [[9], [10], [11]] [[9], [10], [11]] [[9], [10], [11]]. For lumbar spinal stenosis, some studies show that TFESI is more effective than ILESI [12], others found no difference between the two approaches [13], while others have reported a lack benefit from the procedure in patients with degenerative stenosis [14]. Until recently, little work had been done in comparing the TF to the IL approach in the cervical spine, and results have been conflicting. McCormick et al. demonstrated better improvement in “dominant pain” (arm or neck pain) at 1 month, and better improvement in neck pain at 1 and 3 months in the group that received an IL injection with an epidural catheter compared to those who received a cervical TFESI [15]. Lee et al. found no difference in patients with axial cervical pain who received a TFESI compared to those who received an ILESI [16], and Sim et al. demonstrated somewhat better results in patients with cervical radicular pain who received a TFESI compared to those who received an ILESI [17].

Given the paucity and conflicting nature of the literature on this topic, the purpose of our study was to add to this literature by comparing success rates, defined as ≥ 50% improvement in NRS pain scores, of fluoroscopically guided cervical TFESIs vs ILESIs.

## 2 Methods

Institutional Review Board (IRB) approval was obtained at an academic medical center (IRB#48537) for a single site retrospective observational study. A waiver of consent was granted by the IRB. Inclusion criteria were all consecutive patients ≥18 years of age who received a first-time cervical ESI by an interventional spine physiatrist between January 2010 and October 2018. Exclusion criteria included absence of an MRI in the electronic medical record within the 12 months prior to the procedure (an analysis based on MRI findings was planned, but has not yet occurred), and lack of NRS pain score data prior to and within 60 days after the ESI. Additionally, patients who underwent a diagnostic injection without steroid were excluded. Current procedural terminology (CPT) codes 62321, 62310 (cervical/thoracic interlaminar epidural injection) and 64479 (cervical transforaminal epidural injection) were used to search the electronic medical record for eligible patients.

Eligible patients who received cervical ESIs were referred by orthopedic surgeons, neurosurgeons, or physiatrists. All injections were performed by fellowship trained spine physiatrists at a single academic physical medicine and rehabilitation spine center. The technique (TFESI or ILESI), intervertebral level, and when relevant, the side of the injection, were determined by the treating physician based on the clinical evaluation and imaging findings. The injections were performed under fluoroscopic guidance following the Spine Intervention Society Practice Guidelines [18]. All TFESIs were performed using dexamethasone, and all ILESIs were performed using methylprednisolone.

### 2.1 Statistical analysis

Success in the primary outcome was defined as a Numeric Rating Scale (NRS) pain score improvement of ≥50%. The proportion of patients who achieved this response was calculated using baseline and follow-up encounter NRS pain scores, and 95% confidence intervals were calculated. Success rates were compared between patients who received TF and ILESIs. Similar calculations were performed for patients who achieved ≥80% pain relief. Median NRS pain scores with interquartile ranges were calculated. Differences in pre-procedure and post-procedure scores between the TFESI group and the ILESI group were assessed with Mann Whitney U-tests. Differences in the change from pre-procedure to post-procedure scores were assessed similarly. The change from pre-procedure to post-procedure scores within each group was assessed with Wilcoxon signed rank tests. All analyses were performed in SAS v. 9.4 (Cary, NC, USA) with a two-sided level of significance of α ​= ​0.05.

## 3 Results

Overall, 571 patients met search criteria (316 who received a TFESI and 255 who received an IL ESI). A total of 208 patients (138 TFESI and 70 ILESI) were excluded due to lack of follow-up NRS data, follow-up data after 60 days, or no imaging within 1 year. 363 patients remained and were included in the analysis. 178 patients received a cervical TFESI and 185 received an ILESI. There were no differences between baseline pain scores between the two groups (p ​= ​0.104). The median follow-up data collection time for TFESIs was 19 days (range 3 to 60 days), and for ILESIs it was 20 days (range 1 to 60 days). Table 1 shows a list of the distribution of levels of the injections.

**Table 1:** Distribution of levels injected.

Level of Injection	Number of TF injections (Total 178)	Number of IL injections (Total 185)
C2-3	2	0
C3-4	10	0
C4-5	21	0
C5-6	83	0
C6-7	55	3
C7-T1	7	171
T1-T2	0	11

TF ​= ​transforaminal; IL ​= ​interlaminar.

For the primary outcome of ≥50% pain relief, the overall success rate was 52% [95% CI: 47 – 57%]. There was a trend towards better results in patients who received an ILESI with a 59% [95% CI: 52 - 66%] success rate compared to a 46% [95% CI: 39 – 53%] success rate in those who received a TFESI, however the result did not quite reach statistical significance. When evaluating ≥80% pain relief, the ILESI group had statistically better results with a 37% [95% CI: 30 – 44%] success rate compared to a 17% [95% CI: 11 – 23%] success rate in the TFESI group (Table 2.)

**Table 2:** Success rates from cervical TFESI vs ILESI.

	Overall (n ​= ​363)	ILESI (n ​= ​185)	TFESI (n ​= ​178)
≥50% pain relief:	52% [95% CI: 47 – 57%]	59% [95% CI: 52 - 66%]	46% [95% CI: 39 – 53%]
≥80% pain relief:	28% [95% CI: 23 – 33%]	37% [95% CI: 30 – 44%]	17% [95% CI: 11 – 23%]

TFESI ​= ​transforaminal epidural steroid injection; ILESI ​= ​interlaminar epidural steroid injection; 95% CI ​= ​95% confidence interval.

**Fig. 1:**
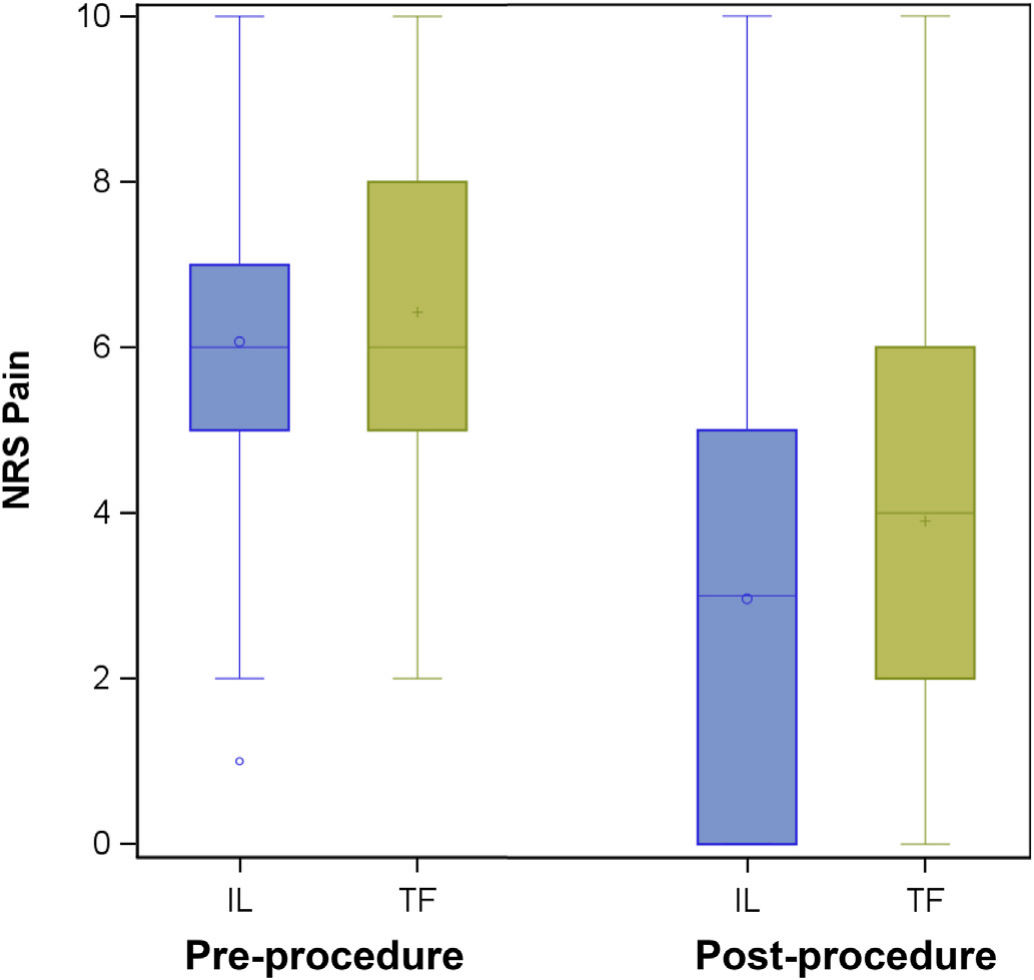
Pre-procedure NRS Scores. Central lines are Medians, Central markers are Means. Edges of box are 1st and 3rd Quartiles. Whiskers go to 1.5∗interquartile range. NRS = Numeric Rating Scale; IL ​= ​interlaminar; TF ​= ​transforaminal.

Post-procedure NRS scores were lower in the ILESI group (p ​< ​0.001). Both groups showed a significant improvement in NRS scores from pre-to post-procedure (p ​< ​0.001 for each group), however the change was greater in the ILESI group (p ​= ​0.040) (Table 3, Fig. 1).

**Table 3:** Change in median pain scores.

	ILESI (n ​= ​185)	TFESI (n ​= ​178)	p-value (difference between groups)
Pre-injection NRS score (median, IQR)	6 (5, 7)	6 (5, 8)	
Post-injection NRS score (median, IQR)	3 (0, 5)	4 (2, 6)	0.040

TFESI ​= ​transforaminal epidural steroid injection; ILESI ​= ​interlaminar epidural steroid injection; NRS ​= ​numeric rating scale; IQR ​= ​interquartile range.

Due to the limited posterior epidural space at higher levels in the cervical spine, the Multisociety Pain Workgroup (MPW) recommends performing interlaminar cervical epidural steroid injections at the C7-T1 level, or not above the C6-7 level [19]. Therefore, the vast majority of our ILESIs were performed at C7-T1, and none were performed above C6-7. In order to compare injection types at similar levels in the spine, we performed a subgroup analysis of the TFESIs performed at the lower levels (C5-6, C6-7, and C7-T1), and found no significant differences in success rates (>50% improvement in NRS) at these levels compared to our findings from the combined TFESI group (43% [95% CI: 32-54%] at C5-6, 44% [95%CI: 31 – 57%] at C6-7, and 43% [95%CI: 6 – 80%] at C7-T1). However, the combined success rate from TFESIs performed between C5-6 and C7-T1 was 43% [95%CI: 35 – 51%], which is significantly lower than the success rate from the ILESIs (59% [95%CI 52 – 66%]).

## 4 Discussion

While our study did not quite meet statistical significance in the primary endpoint (≥50% improvement in NRS), we did demonstrate better success rates (≥80% improvement in NRS), lower median pain scores, and better improvement in median pain scores in patients who underwent cervical ILESIs compared to those who underwent cervical TFESIs. Additionally, our subgroup analysis of cervical TFESIs performed at lower levels (C5-6 through C7-T1) showed a significantly higher success rate in the ILESI group. However, given the retrospective nature of our study, this data cannot be used to make causal inferences about which injection technique is superior. Instead, we encourage additional prospective randomized controlled trials to provide further clarity.

To date, the literature comparing cervical TFESIs to ILESIs is limited and demonstrates conflicting findings including better results from ILESIs in patients with axial and radicular symptoms [15], better results from TFESIs in patients with radicular pain with or without neck pain [17], and no difference between the two techniques in patients with axial cervical pain and/or periscapular pain [16]. Our study adds to this growing body of literature and shows better results in patients who received an ILESI.

Strengths of our study include the fact that this was a large analysis of 363 patients who underwent a cervical ESI at an academic spine center. While the analysis was retrospective, the data was collected prospectively on consecutive patients. Our overall success rate of 52% is similar to that typically seen in the literature [20,21], suggesting that our patient population was similar to those previously studied.

Although our study is one of the first to report on comparative outcomes between cervical ILESIs and TFESIs, it does have several limitations. First, the study was retrospective and was not randomized. Bias likely existed in the selection of which patients received each type of injection. However, the baseline NRS pain scores were similar between the two groups, suggesting that overall pain levels were comparable between the groups. Yet although the pain levels were comparable, our practice patterns may differ for patients selected for a TFESI vs those selected for an ILESI. While we often choose either a TFESI or an ILESI for patients with well-defined, unilateral radicular symptoms from one level pathology, we are more likely to choose an ILESI for patients with less well-defined symptoms and those with multilevel or bilateral pathology. One might suspect that patients with well-defined symptoms from focal pathology would be more likely to benefit from a procedure, thereby favoring the TFESI group in our study. However, this may not necessarily be correct. Since degenerative foraminal stenosis is more common than disc herniations in the cervical spine [22,23], the patients who received a TFESI in our study may in fact have been more likely to be suffering from mechanical compression, and thereby less likely to benefit from a steroid injection. Therefore, the differences in outcomes between the two groups in our retrospective study may have been affected by differences in patient selection between the two groups. Unfortunately, we did not collect demographic information in our retrospective chart review. Similarly, information was not collected regarding the acuity or location of the patients’ symptoms (although it is our practice to perform these procedures on patients with radicular symptoms.) Therefore, readers of this study cannot be certain if the patients included in this analysis are similar to the patients seen in their practices. Another limitation to our study was that only NRS pain data was measured, and details about the pain (axial neck pain vs radicular pain) was not included. Functional outcomes also were not measured. Additionally, we did not collect information on other treatments received by patients between their injection and the follow-up data collection time. Therefore, it is possible that additional treatments were not evenly distributed between the two groups, and this could have potentially affected outcomes. Lastly, our study only reported on short-term follow-up. When successful, epidural steroid injections provide temporary relief from symptoms. The goal of long-term relief following an injection relies on the natural history of the disease process, not the medication in the epidural space. Therefore, long-term follow-up is less relevant.

Finally, all TFESIs in our study were performed using dexamethasone, and all ILESIs were performed using methylprednisolone. While some may opine that the greater success in the ILESI group was due to differences in effectiveness between the steroids that were used, previous work has shown similar outcomes when comparing dexamethasone with particulate steroids during both cervical [24] and lumbar [25,26] TFESIs. Even if particulate steroids did provide better pain relief, consensus opinions from the Multisociety Pain Workgroup (MPW) recommend against the use of particulate steroids in cervical TFESIs [19]. Therefore, the comparison most relevant to most practicing physicians is not particulate vs non-particulate steroid, but instead, the comparison of typical practice patterns - ILESI with particulate steroid vs TFESI with non-particulate steroid. Although an analysis comparing ILESI with non-particulate steroid to TFESI with non-particulate steroid could be performed, the use of non-particulate steroid for ILESIs is not commonplace. Therefore, while such an analysis would be interesting, it would not provide guidance about most practice patterns.

Within the limitations of our study, patients who received a cervical ILESI experienced better results than those who received a cervical TFESI. Additional prospective studies are required to replicate the findings from this study.

The authors report no relevant disclosures or conflicts of interest, and no funding sources.

## Disclosures

No funding sources were provided. The authors all declare no relevant conflicts of interest.

## Funding source

None.

## Funding

IRB approval was obtained at an academic medical center (IRB #48537).

## Declaration of competing interest

The authors declare that they have no known competing financial interests or personal relationships that could have appeared to influence the work reported in this paper.
